# Transcutaneous vagus nerve stimulation for the treatment of depression: a study protocol for a double blinded randomized clinical trial

**DOI:** 10.1186/1472-6882-12-255

**Published:** 2012-12-14

**Authors:** Pei-Jing Rong, Ji-Liang Fang, Li-Ping Wang, Hong Meng, Jun Liu, Ying-ge Ma, Hui Ben, Liang Li, Ru-Peng Liu, Zhan-Xia Huang, Yu-Feng Zhao, Xia Li, Bing Zhu, Jian Kong

**Affiliations:** 1 Institute of Acupuncture and Moxibustion, China Academy of Chinese Medical Sciences, Beijing, 100700, China; 2 Department of Psychiatry, Massachusetts General Hospital, Harvard Medical School, Boston, MA, 02115, USA; 3 Guang An Men Hospital, China Academy of Chinese Medical Sciences, Beijing, 100053, China; 4 Huguosi TCM Hospital, Beijing University of TCM, Beijing, China; 5 Beijing University of Chinese Medicine, Beijing, 100029, China; 6 Clinical Evaluation Center, China Academy of Chinese Medical Sciences, Beijing, 100700, China

**Keywords:** Major depression disorder, Vagus nerve stimulation, Transcutanecous vagus nerve stimulation

## Abstract

**Background:**

Depressive disorders are the most common form of mental disorders in community and health care settings. Unfortunately, the treatment of Major Depressive Disorder (MDD) is far from satisfactory. Vagus nerve stimulation (VNS) is a relatively new and promising physical treatment for depressive disorders. One particularly appealing element of VNS is the long-term benefit in mood regulation. However, because this intervention involves surgery, perioperative risks, and potentially significant side effects, this treatment has been limited to those patients with treatment-resistant depression who have failed medication trials and exhausted established somatic treatments for major depression, due to intolerance or lack of response.

This double-blinded randomized clinical trial aims to overcome these limitations by introducing a novel method of stimulating superficial branches of the vagus nerve on the ear to treat MDD. The rationale is that direct stimulation of the afferent nerve fibers on the ear area with afferent vagus nerve distribution should produce a similar effect as classic VNS in reducing depressive symptoms without the burden of surgical intervention.

**Design:**

One hundred twenty cases (60 males) of volunteer patients with mild and moderate depression will be randomly divided into transcutaneous vagus nerve stimulation group (tVNS) and sham tVNS group. The treatment period lasts 4 months and all clinical and physiological measurements are acquired at the beginning and the end of the treatment period.

**Discussion:**

This study has the potential to significantly extend the application of VNS treatment for MDD and other disorders (including epilepsy, bipolar disorder, and morbid obesity), resulting in direct benefit to the patients suffering from these highly prevalent disorders. In addition, the results of this double-blinded clinical trial will shed new light on our understanding of acupuncture point specificity, and development of methodologies in clinical trials of acupuncture treatment.

**Trials registration:**

Clinical Trials. ChiCTR-TRC-11001201 http://www.chictr.org/cn/

## Background

Major depressive disorder (MDD) is the fourth leading cause of disability worldwide [1], and is projected to become the second leading cause of disability worldwide by the year 2020 [2,3]. Despite the critical need, current treatments for these disorders are far from satisfactory [1,3] due to high non-response rate to treatments, high relapse rates, and frequent intolerable side effects. The etiology and pathogenesis of depression in MDD is not clear; however, it is generally believed that the cause of major depressive disorder is a combination of brain chemistry, family history, and psychosocial environment.

Vagus nerve stimulation (VNS) is a relatively new FDA-approved somatic treatment for treatment-resistant depression (TRD) that can produce significant and clinically meaningful antidepressant effects [1,4-6]. Studies also indicate that VNS may provide long-term sustained benefits [1,5,7], which is particularly compelling given the highly recurrent nature of MDD [3]. However, the involvement of surgery, perioperative risks, and potentially significant side effects have limited this treatment only to those patients who have been treated for depression in the past but have failed to respond to at least 4 prescribed medications and/or established somatic treatment options such as electroconvulsive therapy (ECT) for MDD [8].

This double-blinded randomized clinical trial aims to overcome these limitations of VNS by testing the efficacy of a novel method of transcutaneous vagus nerve stimulation (tVNS) to treat MDD. The rationale for using tVNS is that anatomical studies have shown that the ear is the only place on the surface of the human body where there is afferent vagus nerve distribution [9,10]. Thus, direct stimulation of the afferent nerve fibers on the ear should produce an effect similar to classic VNS in reducing depressive symptoms without the burden of surgical intervention [11].

Additionally, as an important branch of acupuncture, auricular acupuncture has been widely used to treat various disorders including MDD by stimulating points on the ear. Thus, we believe the results of this study will also enhance our understanding of acupuncture mechanisms, shedding new light on acupoint specificity.

## Methods and design

### Trial design

**Figure 1:**
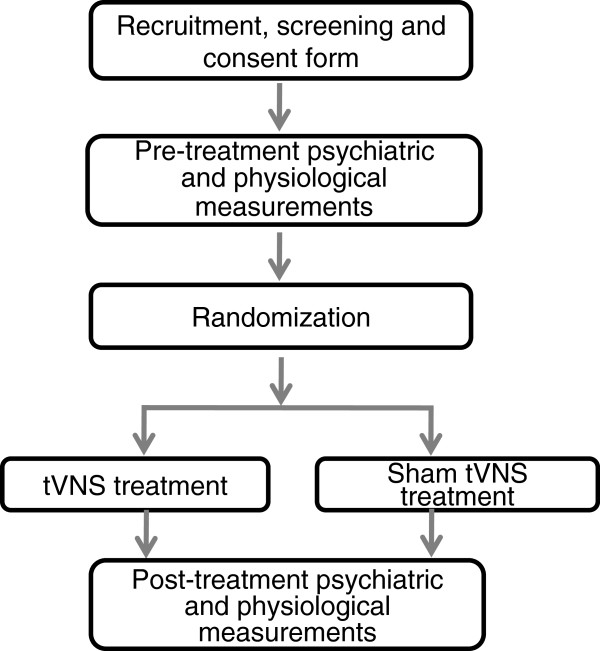
Flow chart of the clinical trial.

This study is a randomized, multicenter, double blind clinical trial with two treatment groups (tVNS and sham tVNS). Please see flow chart (Figure 1) for more details regarding the clinical procedures. The clinical endpoints are assessed by blinded independent observers. The central randomization system is used to assign patients to the tVNS or sham tVNS treatment groups. All procedures are performed by the Clinical Evaluation Center at the China Academy of Chinese Medical Sciences (CACMS) in Beijing. The Ethics committee of Institute of Acupuncture and Moxibustion, CACMS approved the experiment procedure.

### Setting

Investigators are conducting the trial in four hospitals in Beijing, China. These four hospitals are: Guang An Men Hospital, China Academy of Chinese Medical Sciences; Huguosi TCM Hospital, Beijing University of TCM; Acupuncture Hospital, China Academy of Chinese Medical Sciences.

### Blinding

**Figure 2:**
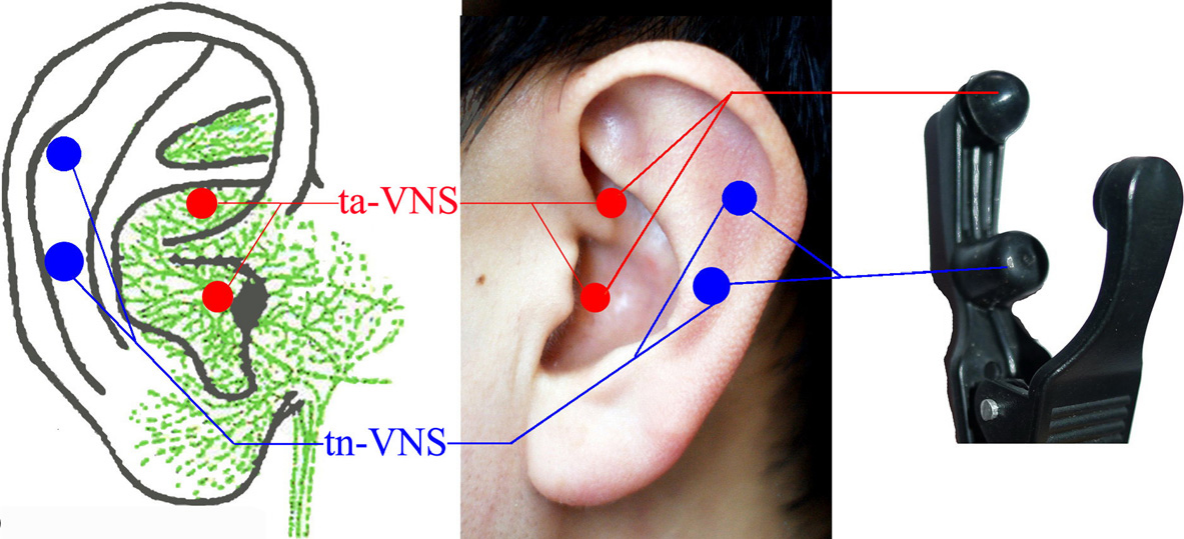
**Locations and the stimulation electrodes on the auricular surface.** Red spots indicate the locations for transcutaneous auricular vagus nerve stimulation (tVNS) and blue spots indicate the locations for sham tVNS. The left figure shows the two tVNS points being in the innervation area of the auricular branch of vagus nerve (green). The right figure indicates the clip/electrode used in the study. To achieve the design of double-blind, two paired electrodes were fixed to one ear clamp. But only one of them was connected with the wire that was imbedded in the clamp in order to keep the operation of double-blind. In the tVNS group, the upper electrodes are wired to the machine while in the sham tVNS group the lower electrodes are wired.

As a double-blinded trial, both study physicians/investigators and patients are blinded to treatment group (tVNS versus sham tVNS). To ensure that both investigators and patients remain blinded, two carbon-impregnated silicone electrodes are fixed to one ear clamp (Figure 2). Only one of the electrodes; however, is connected to the electrical lead (wire) imbedded in the clamp in order to keep the operation of the study double blind. In the tVNS group, the upper electrode was wired to the transcutaneous electrical nerve stimulator (TENS) while in the sham tVNS group the lower electrode is inactively wired to the TENS.

Both the physician’s and the patient’s blindness to the mode of treatment are assessed after completion of all post-treatment measurements by asking each individual to guess the treatment modality they received given three options, “real tVNS”, “sham tVNS” or “uncertain”.

### Patients

#### Study population

Patients with mild or moderate MDD are recruited for the trial. ICD-10 Classification of Mental and Behavioral Disorders are used for diagnosis of MDD. Patients who voluntarily provide informed consent and meet inclusion/exclusion criteria are enrolled in this study. Inclusion and exclusion criteria include:

#### Inclusion criteria

1. Patient meets ICD-10 diagnosis standard: mild (2 typical + 2 other core symptoms), moderate (2 typical+3 other core symptoms).

2. Patient is 16–70 years of age

3. Patient stopped taking anti-depressive medication or other psychiatric medications 2 weeks before the intervention started.

4. Patient is educated beyond junior high school, in order to understand the scales.

5. Patient has exhibited symptoms for at least 2 months, and no longer than 2 years.

#### Exclusion criteria

1. Patients with current addiction to drugs

2. Patients with severe depression or suicidal thoughts

3. Patients with severe medical disorders

4. Patients with poor compliance

### Recruitment procedures

The investigators recruit patients with mild or moderate depressive symptoms using advertising and by sending flyers to the four hospitals involved in the study. After passing a pre-screening, potentially eligible patients provide informed consent in the presence of a study physician.

### Intervention and comparison

#### tVNS treatment

##### Location

The points for tVNS are located in the auricular concha area where there is rich vagus nerve branch distribution (Figure 2).

##### Intervention procedure

Patients take a seated position or they lay on their side. After the stimulation points are disinfected according to standard practice, ear clips are attached to the ear area (auricular concha) that will be stimulated. Stimulation parameters include: 1) density wave adjusted to 20Hz, with less than 1ms wave width; and 2) 1mA current turned on. The intensity is adjusted based on the tolerance of the patient. Each treatment lasts for 30 minutes and is carried on twice a day, 5 days per week for the duration of the treatment period (12 weeks).

#### Sham tVNS treatment

##### Location

The stimulation points for sham tVNS are located at the superior scapha (outer ear margin midpoint), where there is no vagus nerve distribution (Figure 2).

##### Intervention procedure

All procedures performed in the sham tVNS treatment group are identical to the procedures for the verum tVNS group.

### Choice of endpoints

All endpoints are measured at week 0 and week 10. The endpoints include the 24-item Hamilton Depression Rating Scale (HAM-D-24), the 17-item Hamilton Depression Rating Scale (HAM-D-17), Self-rating Anxiety Scale (SAS), Self-rating Depression Scale (SDS), electrocardiogram rate, breathing rate, and skin conduction response. Similar to previous studies [12,13], the primary outcome is the categorical classification of treatment response. We are interested in comparing the difference in treatment response rate between the two groups as measured by HAM-D-24, where treatment response is defined as a 50% or greater reduction in HAM-D-24 scores following a 10-week treatment.

In addition, previous studies [14-17] suggest that expectations for symptom relief can significantly influence the response to medications, acupuncture, and placebo. Thus, before the first treatment, patients are asked to rate on a scale how much they expect the treatment will relieve their symptoms, from “complete relief” to “do not work at all”.

### Sample size calculation and statistical analysis

#### Sample size

We present here our power analysis for the primary outcome only. Since this is a novel therapy, we used the data from a previous non-controlled pilot study on treatment-resistant MDD in senior patients [18] to calculate the power. The primary outcome measure is the categorical classification of response; we define the treatment response as a 50% or greater reduction in HAM-D-24 scores following treatments. From a previous proof of concept study [18], the response rate in the tVNS group is 39%; assuming a 20% dropout rate, with 60 patient in each group, we will have 80% power to detect a difference of 25% or greater in response rate between the tVNS and the sham tVNS group based on a chi square test at a 0.05 significance level.

#### Statistical analysis

In this study, the primary outcome measure is the categorical classification of treatment response. We define the treatment response as a 50% or greater reduction in HAM-D-24 scores following treatments. Response rates across the two groups will be compared with the chi square (*x*^2^) test.

Additionally, we will also use the HAM-D-24 score as continuous variable and apply a regression model to compare the different between two treatment groups. More specifically, in the model, the dependent variable is post-treatment HAM-D-24 score, the independent variable is treatment mode (tVNS versus sham tVNS), and covariances will include pre-treatment HAM score, age and gender. Similar analyses will also be performed in other clinical and physiological outcome measurements.

### Data safety monitoring

Independent data safety monitoring board members will meet every 6 months or as needed. Participants who show persistent worsening symptoms during the course of a clinical trial or develop unstable psychiatric symptoms (e.g., suicidality, homicidality, psychosis) will be withdrawn from the study and will be referred for appropriate treatment immediately.

According to the following classifications, a safety monitoring board will review and rate adverse events to determine whether to suspend the test condition.

Level 1: Security, without any adverse reactions.

Level 2: Safe, and have mild adverse reaction, do not need any treatment can continue to treatment.

Level 3: There are security issues; there is a moderate adverse reaction, after treatment may continue to treatment.

Level 4: Because of adverse reactions, terminate this research.

All adverse events will be reported to the Human Research Committee promptly in accordance with guidelines.

## Discussions

Depression, with serious medical, social and economic consequences, represents a significant burden to both patients and society. Unfortunately, reports suggest that despite the progress that has been made in pharmacologic and psychological treatments, many MDD patients only partially benefit or do not benefit at all [1,3]. Additionally, pharmacologic treatments often have a high rate of relapse and intolerable side effects, which further the call for new treatment of MDD.

VNS is a relatively new FDA-approved somatic treatment for depressive disorders [1,4-6] and may provide long-term sustained benefits [1,5,7]. The limitations and adverse events related to VNS are quite obvious, including the involvement of surgery, perioperative risks, and potentially significant side effects such as hoarseness, throat pain, coughing, dyspnea, paresthesia, and muscle. Thus, VNS treatment is limited only to those patients who have exhausted the standard somatic treatments for MDD due to intolerance or lack of response [8].

The underlying mechanism of antidepressant action using VNS is not fully understood. Hypotheses are based on the anatomy and function of the vagus nerve, which is implicated in mood control [19]. It is known that the vagus nerve is a mixed nerve composed of about 80% afferent fibers. It is speculated that antidepressant effects of VNS are attributed partially to the projection of afferent fibers to the nucleus tractus solitaries, which is further connected directly and indirectly with brain structures including reticular formation in the medulla, parabrachial nucleus, the locus coeruleus, the amygdala, hypothalamus, insula, thalamus, orbitofrontal cortex, and other limbic regions responsible for mood and anxiety regulation [5,9].

This clinical trial aims to overcome these limitations by introducing a novel method of stimulating superficial branches of the vagus nerve to treat MDD. The evidence supporting the feasibility of this trial includes: 1) two proofs of concept, non-controlled, clinical trials demonstrated that tVNS can be used as an effective treatment for treatment-resistant MDD in both senior patients [18] and in epilepsy patients [20], another important indication of the VNS; 2) An fMRI study [21] showed that tVNS at specific ear regions can produce significant fMRI signal decreases, which is similar to the brain activity changes produced by VNS [22-24]. It is important to note that the same stimulation at ear regions without vagal supply could not evoke similar fMRI signal changes. In addition, only after tVNS, psychometric assessment of research subjects revealed significant improvement in well-being; and 3) two animal studies [25,26] demonstrated that stimulation of the certain areas of the ear with vagus nerve supplies can evoke firing of the vagus nerve and produce relatively specific physiological changes (e.g. decreased arterial pressure, heart rate, and intragastric pressure). Thus, results from both human and animal studies have endorsed the rational of the treatments.

In a previous study in senior patients with resistant MDD, Xu and colleagues [18] found that compared with the drug only group, drug plus electroacupuncture stimulation at a point in the ear where the vagus nerve is distributed can produce greater HAM-D score reduction and more good responders. However, the lack of an accepted control group significantly limited the interpretation of the study. In this protocol, we have included an active control group (sham tVNS), and used a double-blinded design, which could significantly enhance the quality of the clinical trial.

Compared with traditional VNS, tVNS has the advantage of being low cost, safe and non-invasive. Thus, it can be used on patients with mild to moderate MDD. If this study is successful, the results will significantly extend the application of VNS treatment to MDD and other disorders (including epilepsy, bipolar disorder, and morbid obesity) and will result in direct benefit to the patients suffering from these highly prevalent disorders.

Additionally, we believe that this study will also enhance our understanding of acupuncture mechanisms. As an important branch of acupuncture treatment and with extensive application in past decades, auricular acupuncture, with specific indication of different ear acupoints, remains a mystery. The tVNS treatment for MDD provides a unique angle as well as a model to investigate the biological basis underlying acupoint specificity.

Finally, to blind both the investigator and patient, we applied two pairs of carbon-impregnated silicone electrodes, only one of which was wired to give electrical output; thus, neither patients nor physicians know whether they received real or sham tVNS. This double-blinded design can significantly improve the quality of the trial and will shed new light on the development of methodologies in clinical trials of acupuncture treatment.

In summary, in this clinical trial, we are evaluating the efficacy of tVNS in mild and moderate MDD patients using a randomized and double-blinded design. The success of the trial will significantly improve the application of this promising new method.

### Trial status

The first participants were included on May 18, 2011. There are 49 participants recruited until the point when this paper was submitted on September, 2012.

## Competing interests

All authors claim no conflict of interest.

## Authors’ contributions

PjR,BZ designed the trail and was responsible for obtaining approval by the Institutional Ethics Committee of the China Academy of Chinese Medical Sciences. PjR ,JL Fang and JK contributed to data analysis plan and manuscript preparation; JlF, LpW, HM, JL, YgM HB, LL RpL, contribute to the design of the trial and are in charge of recruitment and treatment of patients in each center, they also do data collection, LL, RjR, XL prepared the figures and RjR present his ear on Figure 2. and YfZ did central randomization and the protocol of statistical analysis. All authors read the manuscript, and approved the contributions.

## Pre-publication history

The pre-publication history for this paper can be accessed here:

http://www.biomedcentral.com/1472-6882/12/255/prepub
